# The Effects of Cell Phone Waves (900 MHz-GSM Band) on Sperm Parameters and Total Antioxidant Capacity in Rats

**Published:** 2013-03-06

**Authors:** Masoud Ghanbari, Seyed Bagher Mortazavi, Ali Khavanin, Mozafar Khazaei

**Affiliations:** 1 Department of Occupational and Environmental Health, School of Medical Sciences, Tarbiat Modares University, Tehran, Iran; 2 Fertility and Infertility Research Center, Kermanshah University of Medical Sciences, Kermanshah, Iran

**Keywords:** Cell phone wave, Oxidative Stress, Sperm Parameters, Male Rat

## Abstract

**Background:**

There is tremendous concern regarding the possible adverse effects of cell phone microwaves. Contradictory results, however, have been reported for the effects of these waves on the body. In the present study, the effect of cell phone microwaves on sperm parameters and total antioxidant capacity was investigated with regard to the duration of exposure and the frequency of these waves.

**Materials and Methods:**

This experimental study was performed on 28 adult male Wistar rats (200-250 g). The animals were randomly assigned to four groups (n=7): i. control; ii. two-week exposure to cell phone-simulated waves; iii. three-week exposure to cell phonesimulated waves; and iv. two-week exposure to cell phone antenna waves. In all groups, sperm analysis was performed based on standard methods and we determined the mean sperm total antioxidant capacity according to the ferric reducing ability of plasma (FRAP) method. Data were analyzed by one-way ANOVA followed by Tukey’s test using SPSS version 16 software.

**Results:**

The results indicated that sperm viability, motility, and total antioxidant capacity in all exposure groups decreased significantly compared to the control group (p<0.05). Increasing the duration of exposure from 2 to 3 weeks caused a statistically significant decrease in sperm viability and motility (p<0.05).

**Conclusion:**

Exposure to cell phone waves can decrease sperm viability and motility in rats. These waves can also decrease sperm total antioxidant capacity in rats and result in oxidative stress.

## Introduction

Microwaves are part of the wide range of electromagnetic waves with a frequency range of 300 MHz-300 GHz (1). The evidence indicates that these waves are harmful to humans and based on their; intensity, frequency, type, and exposure duration, create biological effects (2). Further, there is tremendous concern for the possible adverse effects of cell phone microwaves. Researchers have warned people of the harmful effects of this radiation on the brain, heart, thyroid, skin, kidneys, eyes, liver, and reproductive tissues, (3-10) although contradictory results have been reported in studies by Dasdag et al. (11), Ferreira et al. (12) and Ahlbom et al. (13).

The Global System for Mobile Communications (GSM) was established in 1987. The majority of European and Asian countries, including Iran, use this system. In GSM, the frequencies transmitted from cell phones to cell phone antennas (base station) range from 870 to 915 MHz (uplink) whereas frequencies transmitted from antennas to cell phones range from 935 to 960 MHz (downlink) (14). The importance of this system and its widespread use is extensive (15); in Iran approximately 37 million people have used cell phones according to GSM in 2010.

There have been few studies on the effects of cell phone waves on sperm parameters. Wdowiak et al. (10) have observed that cell phone waves caused a decrease in motility and percentage of sperm with normal morphology in people who used cell phones. Further, Yan et al. (16) have shown that these waves decreased motility, viability, and the percentage of sperms with normal morphology. A similar study, however, has indicated that increased duration of cell phone use can cause an increase in sperm vulnerability and decrease in sperm parameters (17).

However, the issue in question is that cell phone waves may cause oxidative stress by enhancing lipid peroxidation and changing antioxidant activities in the body (18). Oxidative stress is a process in which the normal balance between per oxidants and antioxidants changes in such a way that leads to strengthening oxidants and biological damage (19).

Antioxidants are molecules responsible for preventing oxidative homeostasis and coping with oxidative stress. These molecules prevent the formation of active oxygen species and inhibit their functions. Antioxidants are classified into two groups: enzymatic and non-enzymatic (20).

Few studies have examined the effects of cell phone waves on antioxidants. The results of a study have indicated that cell phone waves increase lipid peroxidation, decrease the total concentration of thiols and total antioxidant capacity of blood plasma, resulting in oxidative stress (18). Some studies, however, have shown that these waves have no effect on the antioxidant system (21-23).

Sperm are sensitive to oxidative stress. The sperm membrane of mammals is full of unsaturated fatty acids and sensitive to oxidation. Abnormal sperm are responsible for the overproduction of reactive oxygen species (ROS) which result in oxidative stress and considered to be one of the causes of male infertility (24).

Under normal circumstances semen plasma contains sufficient antioxidant mechanisms and is able to neutralize the effect of ROS on sperm. However, if for any reason an imbalance occurs, the sperm goes through changes that negatively influence sperm parameters. Age, environmental factors (e.g., radiation exposure) and nutrition are factors that affect this change (20). De Luliis et al. (25) have observed that cell phone waves decrease sperm mitochondria, motility, and viability through ROS. Sariozkan et al. (26) have indicated that freezing semen to preserve sperm results in lipid peroxidation of the sperm membrane and consequently decreases sperm motility, viability and fertility.

Infertility and its related problems are considered major issues in a couple’s life. The most common cause of infertility in males is their inability to produce sufficient normal, active sperm (24). However, contradictory results have been reported regarding the effects of cell phone waves on sperm (21, 22). This effect, however, is not clear and requires additional investigation. Thus, in the present research we investigate the effect of cell phone waves on sperm parameters. Taking into consideration the importance of antioxidant elements as body protectors (24, 27), we have also evaluated the effect of cell phone waves on sperm total antioxidant capacity.

## Materials and Methods

This experimental research was carried out on 28 male Wistar rats (200-250 g) in the Fertility and Infertility Research Center at Kermanshah University of Medical Sciences. The animals were purchased from Iran Pasteur Institute and kept in the animal house of according to recommended conditions (28) in terms of temperature (21-23˚C), light (12 hours light/12 hours dark), ventilation and food. Recommendations from the Ethical Committee of Tarbiat Modares University was implemented regarding research conducted on the animals.

The rats were randomly assigned to 4 groups of 7 rats in each group according to the study design as follows. Group 1 comprised the control group maintained under experimental conditions, without any exposure to simulated cell phone waves. Group 2 animals were exposed to cell phone receiver simulated waves (915 MHz frequency) for 14 days. Group 3 animals were exposed to cell phone receiver simulated waves (915 MHz frequency) for 21 days and group 4 animals were exposed to simulated waves of a cell phone antenna (950 MHz frequency) for 14 days.

In this study, the 915 MHz frequency represented cell phone receiver waves, whereas the 950 MHz represented cell phone antenna waves. Frequencies of 915 or 950 Hz represented cell phone waves.

### Design and construction of the exposure cylinder and radiation chamber

#### Exposure cylinder

**Fig 1:**
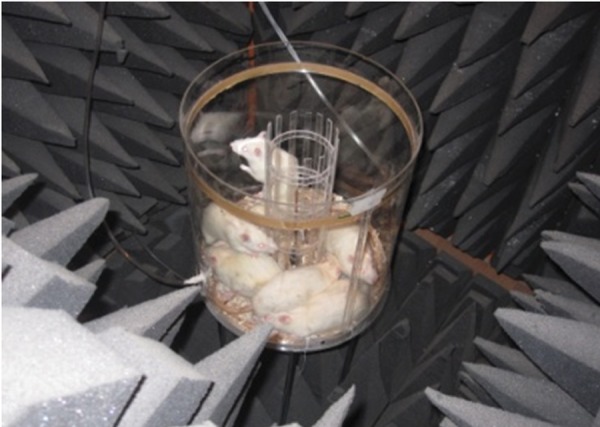
The exposure cylinder and radiation chamber

In order to expose the animals to cell phone-simulated waves, we constructed a Plexi glass cylinder that consisted of internal (radius: 15 cm, height: 30 cm) and an internal (radius: 5 cm, height: 30 cm) cylinder. The animals were placed between the internal and external space during the experiments and had free access to all areas of the space. The internal cylinder was intended to prevent the animals from entering the near field of the monopole antenna which was vertically installed in the center of the internal cylinder. The monopole antenna was used as the simulation device from which cell phone waves (Fig 1) were emitted vertically into the center of the internal cylinder. Animals were prevented from entering this area because measuring the density was not accurate in the field close to the antenna.

#### Exposure to cell phone-simulated waves

The vertical antenna (monopole) of the cell phone simulation generator was placed in the center of the internal cylinder and the density was measured at 5, 10, and 15 cm from the antenna at a height of 5cm from the floor of exposure cylinder using a portable system (Holaday, USA). The average density in the mentioned distances was 1.60 mw/cm^2^.

The rats in groups 2 and 3 were exposed to microwaves (915 MHz) as the carrier wave (switch carrier 217 Hz and modulation 200 KHz) for eight hours a day for 14 and 21consecutive days, respectively. Exposure conditions in group 4 were similar to group 2 except that the frequency of radiation waves was 950 MHz.

#### Measurement of sperm parameters

Animals from all groups were anesthetized with chloroform at the end of the experiment. After opening the anterior wall of the thorax, blood was taken from the heart. The cauda of the left epididymis was separated and segmented in HAM’s/F10 (Gibco, UK) that contained 10% fetal bovine serum which had been maintained at a temperature of 37˚C and 5% CO_2_. After 45 minutes, sperm analysis was performed according to World Health Organization (WHO) instructions (29) as follows.

#### Sperm motility

Sperm motility was examined according to WHO recommendations and categorized as: a. fast progressive; b. slow progressive; c. non-progressive and d. non-motile in ten microscopic fields. The total sperm that comprised categories a and b were determined as the percentage of motility for each sample.

#### Sperm viability

We performed supra vital staining to identify live sperm. One drop of medium that contained sperm was placed on a slide and mixed with a small drop of eosin B (0.5% in saline). The cover slip was immediately placed on the drop and analyzed at ×400 magnification. In this staining method, the head of a dead sperm, due to deficiency in the membrane, absorbs eosin and turns red. However, live sperm do not absorb color. In each slide, 100 sperm were counted and the percentage of viable sperm reported.

#### Sperm counting

A Neubauer hemocytometer was used to count sperm. One drop of a diluted sample was placed on the slide after which all of the sperm in the central square were counted. Sperm count in 1 ml was calculated.

#### Sperm morphology

We used the Papanicolaou staining method to analyze sperm morphology. After staining, sperm in ten microscopic fields were analyzed and classified according to WHO classification as either normal or abnormal (a. deficient in the head, b. deficient in the neck and c. deficient in the cauda). The percentage of sperm with normal morphology was then determined.

After sperm were analyzed they were centrifuged at a temperature of 4˚C for 15 minutes at 2500 rpm. After separating the supernatant, 1 cc of phosphate buffer that contained EDTA (30) was added to the remaining sperm pellet. The samples were preserved at -70˚C until antioxidant measure.

#### Measuring the total antioxidant capacity

The total antioxidant capacity of the rat sperm was measured by the ferric reducing ability of plasma (FRAP) test (Benzie and Strain method) (31). This method is based on the ability of sample to decrease ferric ione (Fe^3+^) to ferro ione (Fe^2+^).

The samples were incubated at 37˚C for 20 minutes, then centrifuged for 10 minutes at 4˚C and 3000 rpm. Using sampler, 850 μl supernatant was separated from the pellet. Then, the sperm from the remaining sperm pellet that contained 150 μl of buffer liquid were broken by sonication (Labsonic, Germany). During sonication, sample were placed in a salt and ice mixture to avoid decreasing the sample’s antioxidant capacity. After the sperm were broken, the sample was centrifuged (4˚C) again at 8000 rpm. Then, the supernatant was separated from the pellet. At the time of preparing the FRAP solution, it was kept in the freezer (4˚C) and measured immediately after the solution was prepared.

#### Ferric reducing ability of plasma (FRAP test)

First, we prepared standard solutions at concentrations of 125, 250, 500 and 1000 μM from FeSO_4_. 7H_2_O. Then, TPTZ powder (0.0247 g) was dissolved in 7.5 ml HCl (40 mM) to prepare the TPTZ solution after which 7.5 ml of an FeCl_3_. 6H_2_O solution (20 mM) and 75 ml of an acetate buffer solution (300 mM, pH=3.6) were added to the TPTZ solution to make the FRAP solution. Chemicals used for the FRAP test were purchased from Merck Company in Germany.

After preparing the FRAP solution, we added 1.5 ml of the solution to 150 μl of distilled water and placed the solution in a water bath (37˚C) equipped with a shaker for five minutes. Then, 50 μl of the experimental or standard group samples were added to the tubes and placed in the same water bath (37˚C) for ten minutes. Immediately the complex absorption rate at a wave length of 593 nm was recorded by spectrophotometer (Jenway 3620D, England). All samples were run in duplicate and measured to enhance analytical accuracy.

#### Statistical analysis

The results are written as mean ± SD. Data were analyzed by one-way ANOVA and the Tukey posthoc test using SPSS software version 16. P<0.05 was considered significant.

## Results

According to the results, the mean sperm viability in the control group was 87.64 ± 1.82%. In the experimental groups the mean sperm viability was 81.14 ± 2.87% (group 2), 74.71 ± 2.80% (group 3) and 81.00 ± 6.61% (group 4) which was a significant decrease compared with the control group. In a comparison between groups, the increased duration of exposure from 2 to 3 weeks resulted in a significant decrease in sperm viability (Table 1). The findings showed that the mean percent of motility in the control group was 49.96 ± 4.59%. In the experimental groups it was 40.91 ± 4.11% (group 2), 32.91 ± 4.09% (group 3) and 41.29 ± 6.41% (group 4), which was a significant decrease in all exposure groups. When the duration of exposure was increased from 2 to 3 weeks, this decrease in motility was statistically significant (p<0.05; Table 1).

For sperm count and normal morphology, we observed no statistical decrease in all exposure groups compared to the control group (p>0.05). A comparison of the exposure groups with each other also showed no statistical difference (p>0.05) in terms of these two parameters (Table 1).

The mean sperm total antioxidant capacity in the control group was 406.35 ± 64.12 μM/60 million sperm and for the experimental groups it was 297.92 ± 92.76 (group 2), 251.16 ± 48.03 (group 3), and 290.34 ± 71.37 (group 4) μM/60 million sperm. A comparison of the total antioxidant capacity in the exposure and control groups indicated that there was a statistically significant decrease in all three exposure groups in terms of sperm total antioxidant capacity. The comparison of exposure groups with each other showed that the mean sperm total antioxidant capacity in group 3 decreased compared with group 2, but this decrease was not statistically significant (p>0.05; Table 2). This comparison, however, between group 2 (915 MHz) and group 4 (950 MHz) revealed no difference. Thus, in terms of sperm total antioxidant capacity, the effect of cell phone waves was similar to that of cell phone antenna waves (Table 2).

**Table 1:** Comparison of the means of sperm parameters in control and exposure groups (one- way ANOVA and Tukey’s test)


Groups	Sperm count (×10^6^) (mean ± SD)	Sperm viability (%) (mean ± SD)	Sperm motility (%) (mean ± SD)	Normal morphology (%) (mean ± SD)

**1**	58.56 ± 6.01	87.64 ± 1.82	49.96 ± 4.59	82.06 ± 4.60
**2**	62.14 ± 8.92	81.14 ± 2.87^a^	40.91 ± 4.11^a^	81.78 ± 3.96
**3**	57.72 ± 8.05	74.71 ± 2.80^bc^	32.91 ± 4.09^bc^	79.70 ± 6.61
**4**	60.19 ± 6.94	81.00 ± 6.61^a^	41.29 ± 6.41^a^	83.37 ± 6.04


Group 1; Control, Group 2; Exposed to simulated cell phone receiver waves (915 MHz) for 14 days, Group 3; Exposed to simulated cell phone receiver waves (915 MHz) for 21 days and Group 4; Exposed to simulated cell phone antenna waves (950 MHz) for 14 days. a; Compared to control group (p<0.05), b; Compared to control group (p<0.001) and c; Compared to group 2 (p<0.05).

**Table 2:** Comparison of the means of total antioxidant capacity of sperm in control and exposure groups (one-way ANOVA and Tukey’s test)


Groups	Total antioxidant capacity μM/60 million sperm (mean ± SD)	P value (vs. control group)

**1**	406.35 ± 64.12	
**2**	297.92 ± 92.76	0.044
**3**	251.16 ± 48.03	0.001
**4**	290.34 ± 71.37	0.025


Group 1; Control, Group 2; Exposed to simulated cell phone receiver waves (915 MHz) for 14 days, Group 3; Exposed to simulated cell phone receiver waves (915 MHz) for 21 days and Group 4: Exposed to simulated cell phone antenna waves (950 MHz) for 14 days.

## Discussion

The present research indicated that significant decreased occurred for sperm viability, motility and total antioxidant capacity in all exposure groups compared with the control group. The comparison between groups showed a significant decrease in percentage of viability and motility with increased duration of exposure from 2 to 3 weeks, but it did not affect sperm total antioxidant capacity. The results of the study were in agreement with the findings of Agarwal et al. (17) which indicated that cell phone waves negatively impacted sperm count, viability and normal morphology and the effects were more serious with increased duration of daily cell phone use. The findings were compatible with the results of a study by Erogul et al. (32) in which cell phone waves decreased sperm motility, but did not affect sperm count.

In a prospective study on 13 males with normal semen analysis, Davoudi et al. (33) found that using GSM phones for hours 6 per day for 5 days decreased rapid progressive motility of sperm which was in accordance with our results. However, it should be noted that the present study found that not only motility but also sperm viability were negatively affected by the use of cell phones.

As mentioned earlier, simulated cell phone waves had no effect on sperm count in rats which contrasted the results by Kesari et al. (34). It should be noted that in their study, samples from humans were used, whereas the current study used laboratory animal samples. Additionally, the differences in duration of exposure in the cited studies should be taken into account.

The results of the current study in terms of the effect of cell phone waves on normal morphology and sperm count contrasted those of other studies. An observational study of 361 males was conducted to determine if there was a correlation between cell phone use and sperm morphology. Males were divided into four groups: i. no use, ii.<2 hours/day, iii. 2-4 hours/day and iv. >4 hours/day. They observed a statistically significant difference in mean normal morphology between the low and high cell phone use group. Fejes et al. showed a significant decrease in sperm count related to cell phone handling frequency (35).

The results of our study have revealed that cell phone waves decrease sperm total antioxidant capacity. A side effect of cell phone waves is increased free radicals, thus our findings can partly be due to the production of these harmful radicals (36).

This finding was in line with another study (37) where cell phone waves increased lipid peroxidation and decreased glutathione antioxidant capacity in the testes and epididymis of rats. The results of our research were also compatible with the findings of a study by Meral et al. (38).

Oxidative stress generated in the testicular organ due to cell phone exposure leads to a build-up of free radicals and ROS levels in sperm. Sperms are susceptible to damage from oxidative stress due to the high content of polyunsaturated fatty acids in their membranes and limited stores of antioxidant enzymes (24).

On the other hand, a decrease in sperm motility and viability is linked to the concentration of superoxide anion in semen. When superoxide is produced extracellulary, it can oxidize membrane phospholipids and cause a decrease in viability (17).

Based on the findings of our study, it was possible that the effect of increased duration of exposure to cell phone waves (from 2 to 3 weeks) on the percentage of motility and viability was more than the increased frequency, and that the time of exposure to cell phone waves might be more important than cell phone frequency. Increased frequency (from 915 to 950 MHz) did not impact the percentage of motility and viability in rats. Similarly, increased frequency did not create a significant difference in sperm total antioxidant capacity in adult male rats.

## Conclusion

It can also be concluded from the findings of this study that cell phone waves may, in addition to affecting sperm parameters, cause oxidative stress in the body and consequently create various diseases. Thus, because of the extensive use of cell phones, further research is required. It is recommended that more attention be paid to cell phone waves as a source of oxidative stress and exposure to these waves be decreased as much as possible. It is also suggested that individuals who spend more time on cell phones be monitored periodically in terms of reproductive system health and it is recommended that they consume a diet full of antioxidants in order to minimize the adverse effects of these waves.
